# Individual Differences and Metacognitive Knowledge of Visual Search Strategy

**DOI:** 10.1371/journal.pone.0027043

**Published:** 2011-10-31

**Authors:** Michael J. Proulx

**Affiliations:** Biological and Experimental Psychology Group, School of Biological and Chemical Sciences, Queen Mary University of London, London, United Kingdom; Nothwestern University, United States of America

## Abstract

A crucial ability for an organism is to orient toward important objects and to ignore temporarily irrelevant objects. Attention provides the perceptual selectivity necessary to filter an overwhelming input of sensory information to allow for efficient object detection. Although much research has examined visual search and the ‘template’ of attentional set that allows for target detection, the behavior of individual subjects often reveals the limits of experimental control of attention. Few studies have examined important aspects such as individual differences and metacognitive strategies. The present study analyzes the data from two visual search experiments for a conjunctively defined target (Proulx, 2007). The data revealed attentional capture blindness, individual differences in search strategies, and a significant rate of metacognitive errors for the assessment of the strategies employed. These results highlight a challenge for visual attention studies to account for individual differences in search behavior and distractibility, and participants that do not (or are unable to) follow instructions.

## Introduction

Recently, cognitive scientists have begun to marshal the methods of individual differences research to understand the variety of inter-individual performance (for a review see [1]). Although simply reporting the average of individual performance has allowed for an explanation of typical human behavior, the goal of individual differences research is to create models of cognition to account for and even predict individual behavior and the functional neuroanatomy of the brain [2].

A crucial ability for an organism is to orient toward important objects and to ignore temporarily irrelevant objects. For a highly visual species such as humans, this requires visual search. Models of visual search have made excellent progress in describing why some search targets are found easily, like a red flower in a grass field, and others are only found with great effort, like a green grasshopper at rest in the grass field [3]. Crucial for these models is an explication of the visual attributes that guide attention [4]. Attention provides the perceptual selectivity necessary to filter an overwhelming input of sensory information to allow for efficient object detection. In the example, the color contrast of the red flower can guide attention rapidly to its location, however the low shape contrast of the grasshopper would not. Attention can be guided by top-down and bottom-up mechanisms [5]. Top-down mechanisms are those that represent the task-relevant attributes that are set by the task instructions or prior experience (such as knowing the color of a flower one wishes to find [6]). Bottom-up mechanisms are those that represent salience in the scene, with local feature contrast the primary determinant of attentional guidance [7]. Being able to explain individual differences in visual search behavior is of great importance as well. For example, many important jobs require visual search experts such as in radiology [8] and in the detection of threats in x-rays of luggage [9]. Furthermore, an ideal model of attention and visual search would be able to account for individual differences in performance that arise from these examples of expertise and those that arise in novices due to other mechanisms.

Differences in working memory capacity have been found to be predictive of visual search performance. For example, Vogel and colleagues have found that an individual's ability to remember a greater number of items using working memory is related to a filtering capacity in visual search that suppresses attentional capture by distracting visual information [10], [11], [12]. This suggests that a greater working memory capacity is related to the ability to not only store a greater number of items in short term memory, but also to encode only those items which are task relevant. Behavioral work in visual search has extended this research to demonstrate that working memory correlates only with top-down visual search performance where task relevance is crucial, but not with bottom-up visual search tasks where salience in the environment guides attention [13]. This work suggests that it might be the individual search strategies that are of primary importance for predicting inter-individual visual search behavior.

Research on eye movement strategies in visual search also supports the idea that search strategies are a crucial determinant of individual search performance. Much of this research has focused on the eye-movement strategies of experts [14], [15]. The eye-movement strategies and the efficacy of instructions have been examined also in non-experts [16], [17]. Boot and colleagues found stable inter-individual differences in eye-movement strategies, however they also reported that the strategies could change as a function of incentive. Other research has shown that incentive, or reward, can serve as a top-down influence on attentional priority, and the eye-movement results in this study are consistent with that [18], [19].

In everyday visual search tasks, observers rarely receive immediate feedback and performance related rewards beyond the satisfaction of having found the target. Furthermore it might not be possible to monitor eye movements in all work that requires visual search. Thus there is a need for a better understanding of individual differences in search strategy in visual search behavior determined covertly by performance (response times and accuracy, such as that carried out previously on correlates with working memory [13]). This has the benefit of contributing to behavioral models of attention and visual search as well has toward developing a means for assessing professional visual searchers [20].

Although much research has examined visual search and the ‘template’ of attentional set that allows for target detection, the behavior of individual subjects often reveals the limits of experimental control of attention. Few studies have examined important aspects such as individual differences and metacognitive strategies. The present study examines individual differences in visual search for a conjunction target [21]. Conjunction search is a good model for natural search behavior as the target of most searches is defined by more than one feature (such as a conjunction of color and shape to find a lemon). Many prominent models of visual search account for conjunction search behavior with an assumption that only top-down mechanisms are used to guide attention because a conjunction search target does not ‘pop-out’ due to bottom-up salience, such as the case where a single red flower is easily detected due to the singleton red object in the scene. Instead one must search for the conjunction of two or more features because neither feature is unique in the scene. In looking for a yellow lemon amongst green limes (same shape, different color) and yellow bananas (same color, different shape), one must look for a conjunction of color and shape to detect the presence of the lemon.

Although it might logically make sense to rely only on top-down processing to find such a conjunction target, a recent study revealed that bottom-up processing is used in conjunction search [22]. This was demonstrated by introducing a third, irrelevant feature (size) to a conjunction search task. The irrelevant size singleton was found to improve detections times when it coincided with the target location, and slowed detection time when it coincided with a nontarget location. A cursory analysis of whether the distractor captured more attention in one nontarget subset (those that matched the color of the target) versus the other subset (those that matched the orientation of the target) revealed that subjects appeared to have a strategy of searching through only one subset of the items, such as scanning only those objects that matched the color of the target. This strategy resulted in the irrelevant feature capturing attention more strongly when it was in that subset rather than the other subset. This subset search strategy had been proposed previously as a standard mechanism in conjunction search [23]. Later researchers, however, proposed it would only arise due to manipulations of the number of items in each subset, such that subjects will search one subset only if it has fewer items than the other [24].

The present study takes the data set from two conjunction search experiments that revealed subject search strategy by using the irrelevant feature of size as a behavioral assay of the target template used by the subjects [22]. That is, the analysis assessed whether each subject was relying primarily on bottom-up processes to guide attention (and thus equally captured by the irrelevant size singleton on all trial types), or on subset search (where attention is capture more when the size singleton coincides with a nontarget in the subset that is being examined). In addition to presenting a thorough analysis of whether the subjects used a particular search strategy in these tasks, the analysis also examines the use of an instructional manipulation, and assesses its efficacy in exhibiting control over the attentional state of the subjects, a crucial issue for models of attention [25]. Finally an analysis of the metacognitive aspects of visual search strategy is carried out as well [26]. That is, the subjects were all debriefed after completing the experiment and asked to describe their self-assessment of their cognitive strategy to carry out the visual search task. This was than compared with the instructions they were provided and the actual performance they exhibited on the search task. These analyses are important both for the basic control of attentional set in visual search or other attentional tasks, as well for the application of this research for improving real-world visual search, where the efficacy of instructions and the accuracy of self report for visual performance are necessary.

## Results

### Experiment 1: Search strategy with standard instructions

The original study [22] reported that subjects relied on bottom-up processing across both subsets (color and orientation) in visual search for a target defined as a conjunction of color and orientation. However it was also noted that a number of subjects appeared to have used a subset strategy. That is, subjects evidently searched for the target in just one subset, such as those that matched the target color, and thus were more distracted by the irrelevant size singleton when it appeared in that subset rather than the relatively ignored subset (orientation). What is not known is whether the subjects were aware of their actual search behavior, and whether the accuracy of their metacognitive state has any impact on their ability to carry out the search task effectively.

**Figure 1:**
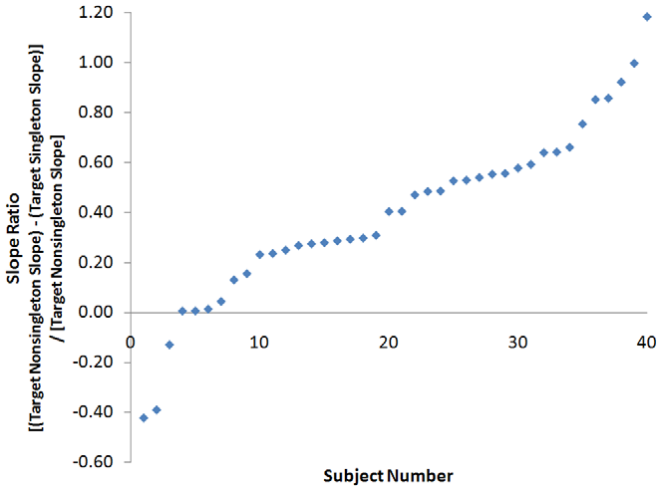
The magnitude of attentional capture in Experiment 1, taking into account task difficulty, through calculation of the slope ratio (subtracted the Target Singleton Slope from the Target Nonsingleton slope, and divided by the Target Nonsingleton slope), represented as a proportion on the y-axis and rank ordered by individual subjects on the x-axis.

First, all subjects were asked whether the irrelevant size singleton distracted them during search for a color-orientation target. All subjects (n = 40) reported that the size singleton was not distracting. This can be contrasted with the finding that only a small minority of subjects (n = 6) were accurate in this assessment and did not exhibit any level of attentional capture by the size singleton (see Figure 1). The magnitude of attentional capture is best expressed in terms of the slope ratio calculated by subtracting the slope of the target singleton trials (when the size singleton coincided with the target) from the slope of the target nonsingleton trials (when the size singleton coincided with a nontarget in either subset, but the target was still present in the display), and dividing by the nonsingleton slope. This measure takes into account both the strength of the stimulus that captures attention and the difficulty of the task [27]. Thus 34 out of 40 subjects in that experiment were distracted by the size singleton to various degrees, three responded with equal efficiency on target present trials no matter where the singleton was located, and three appeared to inhibit the location of the size singleton to such a degree that they responded faster on average when the target was not the largest item, than when it was the largest (and thus had a negative slope ratio, the other three had a slope ratio of zero). Interestingly a split-half analysis of the even versus odd trials for these three subjects that appeared to inhibit the size singleton in fact demonstrated a mixture of a negative slope ratio on half of the trials and a positive slope ratio on the other half. Thus these six subjects were primarily distinct due to not exhibiting distraction by the size singleton compared to the other 34 subjects.

Second, the subjects were asked to classify and describe their search strategy. As already noted, only six were correct in identifying that the singleton did not capture attention. Of these six, three stated that the target was the odd one out, and the other three stated that they searched the color subset. Given that performance was essentially at ceiling for these subjects and there was no attentional capture it is unclear whether this is the case on the basis of behavioral data alone.

Amongst the remaining 34 subjects, they reported that they either searched primarily amongst the color subset (n = 21), the orientation subset (n = 3), non-subset selective search of all items (n = 1), that the target simply ‘popped-out’ of the display (n = 5), or that a serial search was made of the items until the target was found (n = 2). The conjunction search task is a difficult search where the target does not generally pop-out and thus has an appearance of serial search. If some of the subjects were experiencing pop-out or serial search, then the different target present trials types that relate to the location of the size singleton should not be significantly different, however none of these subjects (n = 7) were among those who were unaffected by the size singleton.

**Figure 2:**
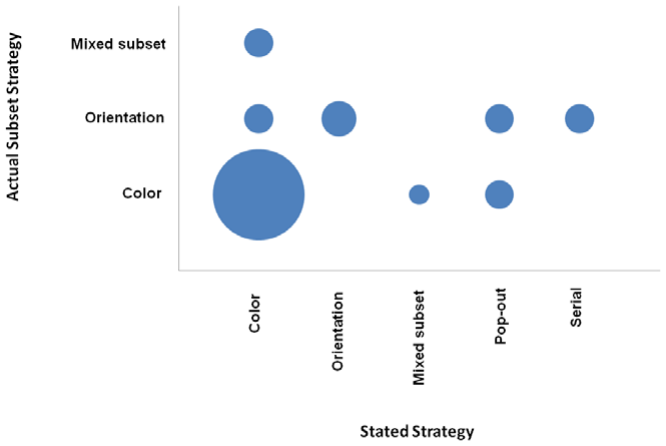
Bubble chart representing metacognitive accuracy in Experiment 1. This depicts the number of subjects by the actual strategy employed (as revealed by the response time data) as a function of the strategy that each subject stated was used. Note that the bubble size is directly proportional to the number of subjects represented (e.g., in this chart, 21 subjects for Color x Color; 1 subject for Mixed subset x Color).

Thus all subjects that exhibited some level of attentional capture (n = 34) were examined further in terms of their reported strategy and under what conditions the singleton captured attention, keeping in mind that the other six subjects were accurate in their assessment of not experiencing attentional capture. Figure 2 displays the reported strategy that was used by summing the columns of disks that correspond with the strategies depicted on the horizontal axis. The slopes for the nontarget-trial types were compared to see if the subjects maintained the same strategy, which would be indicated by having one slope (e.g., the nontarget-color slope) greater than the other slope (e.g., the nontarget-orientation slope). The difference between the slopes (nontarget-color minus nontarget-orientation) resulted in a score for each subject, which allowed a classification of when the singleton captured attention (in the color subset, orientation subset, or non-selective). The diagonal running from lower left to upper right reveals those subjects that were accurate in assessing their own search strategy (n = 23) in terms of the singleton capturing attention more often when it was in the subset that was being attended. The remainder either unintentionally searched the color subset, the orientation subset, or was not subset selective and thus experienced attentional capture when the size singleton appeared in either subset.

In this experiment, approximately 32% of the subjects whose attention was captured were distracted by the singleton even when it should have been less distracting due to their stated strategy describing the phenomenology of the experience. The possible consequences of this metacognitive error were explored by examining the overall response times for subjects as a function of metacognitive accuracy. There was a trend suggestive that those committing metacognitive errors, and thus inaccurate in reporting their search strategy, had faster overall response times than accurate subjects, however this difference was not statistically significant (repeated measures ANOVA with trial type and group as factors, *F*<1.5).

### Experiment 2: Search strategy with subset search instructions

The first experiment provided an assessment of the individual differences for metacognitive search strategies in a conjunction search task with standard instructions. An irrelevant feature was added to the display (size) to assess the success of the search strategies employed. Many search tasks, such as scanning x-ray images of luggage for threats, are accompanied by either explicit instructions or implicit instructions via a training protocol [20], [25]. Furthermore most visual search studies employ instructions to set the target template for the task and models of visual search also generally assume those instructions will be followed, and serve as the top-down input to guide attention [3], [28]. This experiment assessed whether subjects would and could heed instructions to search specifically through only one subset (color or orientation) to find the target, as prior research has suggested this might be more efficient by essentially halving the set size that must be scrutinized for the target [23].

**Figure 3:**
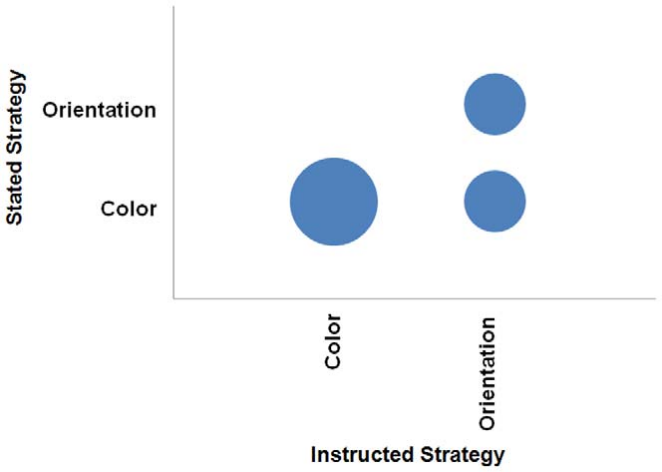
Bubble chart representing the stated adherence to instructions in Experiment 2. This depicts the number of subjects that stated a particular strategy as a function of the instructed strategy condition to which they were randomly assigned.

The subjects were asked to report the actual strategy they used, independent of the instructions they received. Figure 3 presents the reported strategy that subject stated they actually used in the experiment as a function of the instructions they were given. This reveals that although 100% of the subjects in the color-instruction condition reported that they followed the instructions, 50% of the subjects in the orientation-instruction condition reported that they attended to the color subset instead. It was mentioned by the subjects that they found it easier to attend to color rather than orientation. It is interesting to compare Figure 3 with Figure 2. The number of stated strategies were fewer in this case, suggesting that the change in the instructions constrained how the subjects either strategically approached the task or how they conceived of their own strategy.

**Figure 4:**
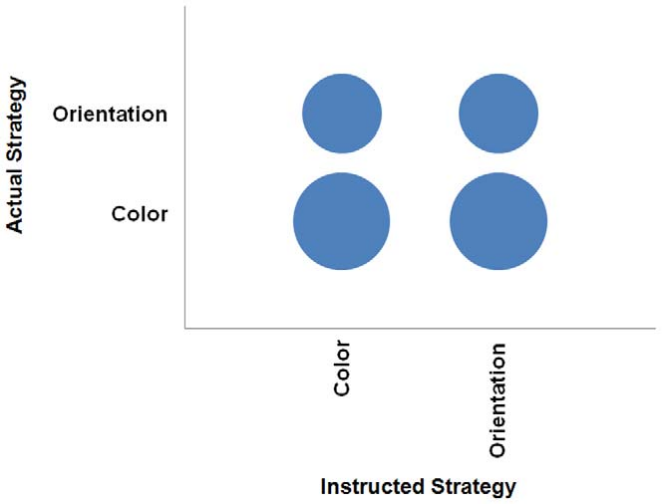
Bubble chart representing the actual instructional adherence in Experiment 2. This depicts the number of subjects by the actual strategy employed (as revealed by the response time data) as a function of each instructed strategy condition to which they were randomly assigned. The bubble size is directly proportional to the number of subjects represented.

Next the slopes for the nontarget trials were compared to determine whether the size singleton was more distracting when in one subset rather than the other as a function of the stated strategy of the subjects. The results of this classification are shown in Figure 4. The diagonal includes those subjects whose actual performance suggests they followed instructions, which was only half of the subjects. There was a bias towards searching the color subset rather than the orientation subset, consistent with comments that attending to that subset was easier. However it is also important to note that 40% of those instructed to attend to the color subset may not have done so, and attended to the orientation subset instead due to the increased distraction of the size singleton when it was in the orientation subset. This suggests that, counter to reported metacognitive awareness, not all subjects necessarily thought the color subset was simpler to attend to given that some in that instructional condition switched subsets as well.

**Figure 5:**
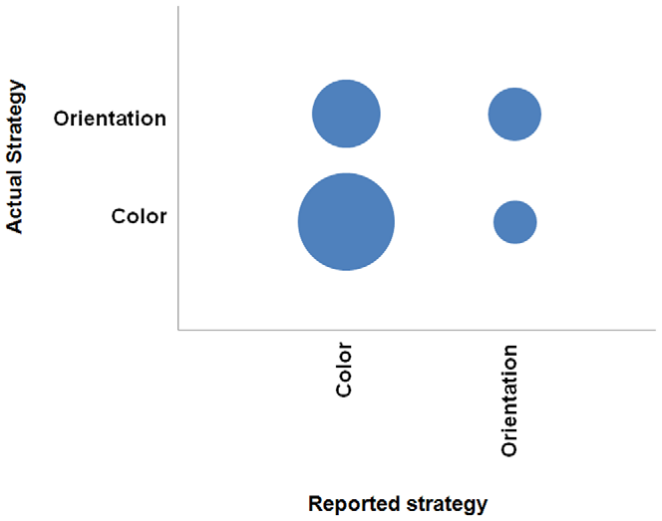
Bubble chart representing metacognitive accuracy in Experiment 2. This depicts the number of subjects by the actual strategy employed (as revealed by the response time data) as a function of the strategy that each subject stated was used.

The analysis of the actual instructional condition versus when the singleton was most distracting does not reveal, however, the metacognitive nature of the strategy employed by the subjects. How accurate were the phenomenological impressions of the subjects for their own experience? Figure 5 reveals when the singleton most distracted the subjects as a function of their reported strategy. Again the diagonal reveals those cases where distraction and strategy match; this suggests that only 13 out of 20 subjects accurately assessed their performance. The performance consequences of this metacognitive error were examined also. As with the data from the first experiment, there was a trend suggestive that those who were inaccurate in reporting their search strategy had faster overall response times than accurate subjects, however this difference was not statistically significant (repeated measures ANOVA with trial type and group as factors, *F*<2).

## Discussion

This study of the metacognition and individual differences of search strategy reveals that awareness of attentional capture might be nonexistent, that not all subjects are aware of the search strategy that is actually employed, and that subjects unintentionally and intentionally do not necessarily follow instructions. These results have several important implications for research on visual search, attention, and the application of such research in real-world settings.

The finding that none of the subjects were aware of the attention-capturing power of the size singleton suggests that there is a form of ‘attentional capture blindness’ at work in visual search. This extends previous notes in the literature. Most and colleagues described attentional capture research as an implicit measure because awareness of the feature that captures attention is not explicitly tested; instead the impact of that feature on another task (detecting a target) reveals whether or not the feature captured attention [29]. For example, Yantis reported in a footnote that the abrupt onset of new objects in their study (which is no longer conspicuous after it has onset) was reported by some subjects as not being noticed [30]. Interestingly, Kramer and colleagues reported in an eye-tracking study that a bright onset was more likely to draw overt attention in older adult subjects if they were aware of the onset than if they were not aware of it [31]. In contrast here, however, it was found that when subjects were specifically asked whether the irrelevant feature captured their attention (all mentioned noticing the size singleton, which remained conspicuous unlike in prior studies [30], [31]), and thus distracted the subjects from the primary task, none of the subjects reported that it did. Certainly this might also be a case of ‘attentional capture amnesia’ and future work assessing the reason behind this metacognitive error would be of interest. This finding also draws interesting parallels with a study that examined awareness of contingent capture, that is attentional capture mediated by the attentional set of the subject. Kawahara [32] reported that although 33 out of 37 subjects thought they were searching for a particular feature, such as a particular color, only 6 actually were doing so (see Kawahara's Table 1). This was demonstrated by finding that those 6 had their attention captured only when the singleton matched that target color; the other 27 subjects had their attention captured by any color, indicating that they were instead actually searching for any unique singleton in the display. The subjects in Kawahara's study were therefore unaware of the degree to which they experienced attentional capture.

Both experiments also revealed that 32–40% of the subjects are unaware of the strategy they employ to detect the target as revealed by when the singleton captured attention. This suggests that self-reported search strategies would not provide a firm basis for the assessment of successful search styles in applied situations [8], [20]. There was no clear difference in performance between those who were able to accurately assess their search strategy and those who were inaccurate, consistent with past research on awareness of attentional orienting [26]. There was a trend in the data that surprisingly suggests that those who make this metacognitive error are possibly faster overall than those who are aware of their search strategy, however further research will be necessary to assess this. More importantly, recent research has revealed the trial-by-trial fluctuations that can occur as the attention state of the subject can vary throughout an experiment [33], and clearly future experiments should also assess whether subject search strategies vary in a similar fashion and be constrained by instructions.

Finally, and of great importance for models of attention [25], basic research on visual search, and for applied searches was the finding that many subjects are distracted by the singleton even when that should have been prevented by search strategy instructions. In fact, the tendency of participants to rely on bottom-up processing in the additional singleton search paradigm employed by Theeuwes [34] might be interpreted as a classic example of this issue when interpreted by the top-down perspective of Bacon and Egeth [35]. The practical problem of not being able to instantiate a particular attentional set in subjects through instructions has been noted before by Bacon and Egeth [36], who suggested bottom-up manipulations to direct the subjects attention such as manipulations of the size of each subset in conjunction search. Bacon and Egeth also incorporated misleading instructions to countermand the problem, and it would be interesting in future work to see if such a manipulation could work here as well. In the present study, only half of the subjects followed instructions, when given one subset or the other to scan and find the target within. Previous research suggests that this would be an efficient strategy given that the effective set size to be examined would be halved [23]. Despite that incentive, half of the subjects switched to the other subset than that instructed. Other studies have revealed more successful attempts at having subjects follow instructions through the use of trial-by-trial feedback and monetary awards in an eye-tracking experiment [17]. However it is also important to note that it might not be possible under all real-world conditions to provide such incentives, and certainly trial-by-trial feedback might even be impossible in fields such as radiology. Another interesting question for further research would be whether subjects would more accurately report their strategy on a trial-by-trial basis. This would provide an important assessment of whether to classify these findings as a case of attentional capture “blindness” or “amnesia.” It is certainly possible that part of the metacognitive inaccuracy could arise from the assessment after completing the experiment, however this study on the topic is useful in providing an initial report from a standard visual search experiment in which the subjects were unaware of this aspect of the study and thus should have exhibited standard behavior during the experiment. It is possible that an online measure of awareness would also lead to changes in visual search behavior.

There is a challenge for visual search models and training protocols to account for individual differences in search behavior and distractibility, participants that do not (or are unable to) follow instructions, and participants that are not necessarily aware of what they are doing. A combination of methodological controls and post-hoc categorization of participant behavior might be necessary to best predict the mental and neural correlates of attentional set in visual search, and further examinations of the correlates of individual performance in visual search will be useful.

The top-down, target template employed by subjects must be known to accurately model attention and determine the neural correlates of attentional control. For example, studies that have examined the neural correlates of conjunction search should take the control of search strategy and individual differences into account. A recent fMRI study of conjunction search sought to constrain the strategies available to subjects by modifying the search task. Leonards and colleagues [37] modified a feature search task for an orientation singleton target to include nontargets of different colors so that the task would more closely resemble a conjunction search task. In addition, they modified a conjunction search task by having the target's defining features change from trial to trial (see also [38], [39]). This modification was made to discourage subjects from restricting search to one subset (see e.g., [23]), which would otherwise make the conjunction task too similar to the feature search task. However the fact that subjects would not know what the target was on any given trial meant that they had to rely on bottom-up processing to find the target (such as searching within each subset until the target was detected). The fact that this study found highly overlapping networks of brain areas involved in both efficient and inefficient search might be explained by the shared bottom-up search strategy used in both tasks. The only brain region that was more active in inefficient versus efficient tasks was an area of superior prefrontal cortex, that previous research has associated with working memory [40]. This result might be telling, because this region is thought to be specifically involved in spatial working memory (see also, [41]). Without knowing in advance what the target's defining features are, it is possible that subjects had to use some working-memory mechanism to assist with maintaining spatial information about each subset while selectively searching each subset for the unknown target.

A number of recent studies have reported that individual differences in working memory can predict attentional control, distraction, and visual search performance [10], [11], [12], [13]. Working memory has been hypothesized to serve as the mechanism for maintaining a top-down target template [28] and future work examining the intersection between attentional capture, metacognitive search strategies, and working memory should prove to be enlightening for basic and applied visual search research.

## Materials and Methods

The standard visual search data from both experiments were reported previously, however all of the analyses of that data set presented here are novel [22]. The basic methods are reported below, and the full details of the experiments and the original data analysis are available in the original study.

### Ethics Statement

All subjects participated either in partial fulfillment of a course requirement or for payment after giving written informed consent. All experiments were conducted under the tenets of the Declaration of Helsinki and received Johns Hopkins University Institutional Review Board approval.

### Experiment 1

Subjects were 40 undergraduates reporting normal or corrected-to-normal vision. All gave informed consent and took part either for payment or for a course requirement.

Subjects were 55 cm from the screen and used a chin rest in a dimly lit room. Stimuli were presented by a C++ and OpenGL program on an IBM-compatible computer. Bars were either blue or green and either right-tilted (45°) or left-tilted (−45°). The nonsingleton bar size subtended 0.6° of visual angle in length and 0.15° in width. The size singleton bar subtended 0.9° in length and 0.15° in width. There was no fixation point and the background was black. A size singleton was present on every trial.

Subjects were randomly assigned to one of four feature-assignment groups (10 per condition) that each had a different set of features assigned to the target or the nontargets: (a) Group A, target was blue and right-tilted (and nontargets were either green and right-tilted or blue and left-tilted); (b) Group B, target was blue and left-tilted; (c) Group C, target was green and right-tilted; (d) Group D, target was green and left-tilted. There was no effect of feature-assignment group [22].

The size singleton appeared on each trial, and coincided with the target on 1/*d* of the trials, where *d* is the number of elements in the display. The size singleton coincided equally often with each nontarget-type on the remainder of the trials. Subjects were instructed to look for the particular features that defined the target for their condition and were informed of the 1/*d* relationship between the size singleton and the target. A display of bars appeared on each trial and the subject responded present or absent with a key press. Errors were signaled with auditory feedback. Each trial began after a two-second inter-trial interval. Each subject participated in 2 blocks of 270 trials per block. Each block included an equal number of target absent and target present trials, and an equal number of trials for each set size. Order of trial types was randomized. Subjects began with a practice block of 20 trials.

### Experiment 2

This experiment attempted to have all subjects engage in a strategy of searching within the orientation subset (n = 10) or the color subset (n = 10) by giving them instructions to do so. All subjects searched for a target that was green and right-tilted (45°). Nontargets were green and left-tilted (−45°), or blue and right-tilted. Subjects were randomly assigned to one of two conditions (10 per condition), with a different set of instructions for each condition: (a) subjects were instructed to search for the target among the color (green) subset; (b) subjects were instructed to search for the target among the orientation (right-tilted) subset.

### Self assessment of search strategy

All subjects were debriefed after completing the experiment. First the subjects were asked whether they thought the size singleton captured their attention and was distracting throughout the experiment, with a response of yes or no. Next the subjects were asked which strategy they used to carry out the task in an open-ended fashion. These responses were coded into the categories on the x-axes of Figures 2 and 3 by a research assistant that was unaware of the purpose of this question and subsequently checked by the author, with no changes to the coding.
